# International trends in the uptake of cancer risk reduction strategies in women with a *BRCA1* or *BRCA2* mutation

**DOI:** 10.1038/s41416-019-0446-1

**Published:** 2019-04-11

**Authors:** Kelly Metcalfe, Andrea Eisen, Leigha Senter, Susan Armel, Louise Bordeleau, Wendy S. Meschino, Tuya Pal, Henry T. Lynch, Nadine M. Tung, Ava Kwong, Peter Ainsworth, Beth Karlan, Pal Moller, Charis Eng, Jeffrey N. Weitzel, Ping Sun, Jan Lubinski, Steven A. Narod, the Hereditary Breast Cancer Clinical Study Group

**Affiliations:** 1 Women’s College Research Institute, Toronto, ON Canada; 2 Bloomberg, Faculty of Nursing, University of Toronto, Toronto, ON Canada; 3 Toronto-Sunnybrook Regional Cancer Center, Toronto, ON Canada; 4 Division of Human Genetics, The Ohio State University Medical Center, Comprehensive Cancer Center, Columbus, OH USA; 5 Division of Gynecologic Oncology, Department of Obstetrics and Gynecology, University of Toronto, Toronto, ON Canada; 6 Juravinksi Cancer Centre, Hamilton, ON L8V 5C2 Canada; 7 North York General Hospital, Toronto, ON Canada; 8 Vanderbilt-Ingram Cancer Center/Vanderbilt University Medical Center, Nashville, TN USA; 9 Hereditary Cancer Center, Creighton University School of Medicine, Omaha, NE USA; 10 Beth Israel Deaconess Medical Center, Boston, MA USA; 11 Department of Surgery, The University of Hong Kong, Queen Mary Hospital, Pokfulam, Hong Kong SAR; 12 Department of Surgery, Hong Kong Sanatorium & Hospital, Happy Valley, Hong Kong SAR; 13 Hong Kong Hereditary Breast Cancer Family Registry, Happy Valley, Hong Kong SAR; 14 Department of Population Sciences, Beckman Research Institute of City of Hope, Duarte, CA USA; 15 Department of Obstetrics and Gynecology, Cedars-Sinai Medical Center, West Hollywood, CA USA; 16 Research Group Inherited Cancer, Department of Medical, Genetics, Oslo University Hospital, Oslo, Norway; 17 Department of Tumor Biology, Institute of Cancer Research, The Norwegian Radium Hospital, part of Oslo University Hospital, Oslo, Norway; 18 Center for Hereditary Tumors, HELIOS-Klinikum Wuppertal, University of Witten-Herdecke, Wuppertal, Germany; 19 Genomic Medicine Institute, Center for Personalised Genetic Healthcare, Cleveland Clinic, Cleveland, OH USA; 20 City of Hope National Medical Center, Duarte, CA USA; 21 International Hereditary Cancer Center, Department of Genetics and Pathology, Pomeranian Medical University, Szczecin, Poland; 22 Dalla Lana School of Public Health, University of Toronto, Toronto, ON Canada

**Keywords:** Cancer prevention, Breast cancer

## Abstract

**Background:**

Women with a *BRCA1* or *BRCA2* mutation face high risks of breast and ovarian cancer. In the current study, we report on uptake of cancer screening and risk-reduction options in a cohort of *BRCA* mutation carriers from ten countries over two time periods (1995 to 2008 and 2009 to 2017).

**Methods:**

Eligible subjects were identified from an international database of female *BRCA* mutation carriers and included women from 59 centres from ten countries. Subjects completed a questionnaire at the time of genetic testing, which included past use of cancer prevention options and screening tests. Biennial follow-up questionnaires were administered.

**Results:**

Six-thousand two-hundred and twenty-three women were followed for a mean of 7.5 years. The mean age at last follow-up was 52.1 years (27–96 years) and 42.3% of the women had a prior diagnosis of breast cancer. In all, 27.8% had a prophylactic bilateral mastectomy and  64.7% had a BSO. Screening with breast MRI increased from 70% before 2009 to 81% at or after 2009. There were significant differences in uptake of all options by country.

**Conclusion:**

For women who received genetic testing more recently, uptake of prophylactic mastectomy and breast MRI is significantly higher than those who received genetic testing more than 10 years ago. However, uptake of both BSO and breast MRI is not optimal, and interventions to increase uptake are needed.

## Background

Women with a *BRCA1* or *BRCA2* mutation face elevated risks of breast and ovarian cancer. The risk for breast cancer to age 80 is 72% for *BRCA1* mutation carriers, and 69% for *BRCA2* mutation carriers; the risk for ovarian cancer is 44% for *BRCA1* carriers and 17% for *BRCA2* carriers.^1^ Several surveillance and prevention options are available with the goals of early detection and of reducing cancer incidence and mortality. The National Comprehensive Cancer Network (NCCN) guidelines state that women with a *BRCA* mutation should receive annual breast MRI and should have bilateral salpingo-oophorectomy (BSO) by the age of 40 years,^2^ BSO has been shown to reduce ovarian cancer incidence and all-cause mortality.^3,4^ Breast screening using magnetic resonance imaging (MRI) is also recommended for *BRCA* mutation carriers. The sensitivity of MRI exceeds that of mammography and MRI screening has been shown to downstage breast cancer,^5–12^ and there is some preliminary evidence that MRI combined with annual mammography may offer a survival advantage in BRCA2 carriers.^13,14^ Another option for women with a *BRCA* mutation is bilateral prophylactic mastectomy, which has been shown to significantly reduce breast cancer incidence in women with a *BRCA* mutation,^15–19^ but studies of prophylactic mastectomy reducing mortality are forthcoming.

In 2008, we reported on the uptake of cancer screening and of various prevention options (surgery and chemoprevention) in 2677 women with a *BRCA* mutation from nine countries.^20^ There were significant differences in the uptake of prophylactic mastectomies and oophorectomies, and breast screening by country. Since this initial report, there is increasing evidence of the beneficial impact of preventive surgeries on cancer incidence and mortality and MRI screening has become a standard of care. In the current study, we report on uptake of cancer screening and risk-reduction options in an expanded cohort of *BRCA* mutation carriers from ten countries, and estimate the uptake rates among those who received their genetic test before and after our initial report in 2008.

## Methods

### Study population

Eligible subjects were identified from an international database of female *BRCA1* and *BRCA2* mutation carriers and included women from 59 centres from ten countries (Austria, Canada, China, France, Israel, Italy, Norway, Holland, Poland and USA). The study received ethics approval from all participating centres, and all study subjects provided written informed consent.

Subjects were eligible for this study if they were known to be a *BRCA1* or *BRCA2* mutation carrier, were between 25 and 80-years-old, and had no prior history of cancer, other than breast cancer, before the baseline questionnaire. Subjects who had been diagnosed with unilateral breast cancer prior to genetic testing were included. Women who were diagnosed with breast cancer during the follow-up period were excluded. All subjects had a minimum of 18 months of follow-up after genetic testing and were alive at the date of follow-up.

### Procedures

Subjects completed a baseline questionnaire at the time of genetic testing, which included demographic information, cancer history, and past use of cancer prevention options and screening tests. Biennial follow-up questionnaires were administered by telephone or by mail. Questions assessed uptake of various cancer preventive options, including prophylactic surgery (mastectomy or oophorectomy), chemoprevention (tamoxifen or raloxifene), breast screening (mammography, MRI) and new cancer diagnoses.

### Statistical analysis

We compared the frequency of various interventions by country. We also compared the frequency of interventions before 2009 and at or after 2009, which corresponds to the cutoff date of patients included in our previous paper. The chi-square test was used to compare frequencies of categorical variables and ANOVA was used to compare the mean values of continuous variables among different regions. All Statistical tests were done by statistical software SAS version 9.1.3, SAS Institute, Inc., Cary, NC, USA. For six countries (Austria, Israel, France, Italy Holland and Norway) data was only available for the period prior to 2009 and these countries did not contribute information for the second period.

## Results

Ten-thousand seven-hundred and ninteen women were identified with a *BRCA1* or *BRCA2* mutation. We excluded 4496 women; 615 women were <25 years at baseline, 54 women were older than 80 years at baseline, 1767 women had cancer other than breast cancer at baseline, 1039 women had no follow-up, 192 women had <1.5 years of follow-up, and 829 were deceased by time of first follow-up. Of these  6223  women met eligibility criteria described above and were included in the analysis.

The mean time of follow-up from time of genetic testing (baseline questionnaire) to last follow-up questionnaire was 7.5 years (range 1.5–22.2 years). The mean age of the participants at last follow-up was 52.1 years (range 27–96 years). 2634 (42.3%) women had a prior diagnosis of unilateral breast cancer (Table 1).

**Table 1:** Characteristics of 6223 mutation carriers country

Variables	Austria	Canada	China	France	Israel	Italy	Holland	Norway	Poland	USA	All	*P*-value^a^
	N = 127	N = 1780	N = 30	N = 30	N = 193	N = 42	N = 85	N = 408	N = 2054	N = 1474	N = 6223	
	(2.0%)	(28.4%)	(0.5%)	(0.5%)	(3.1%)	(0.7%)	(1.4%)	(6.5%)	(32.8%)	(23.6%)	(100%)	
Mutation												
Number (%)												
* BRCA1*	98(77.2)	957(53.8)	11(36.7)	25(83.3)	116(60.6)	32(76.2)	67(78.8)	330(80.9)	2029(98.8)	903(61.3)	4568(73.4)	<10^−4^
* BRCA2*	28(22.1)	790(44.4)	19(63.3)	5(16.7)	65(33.7)	10(23.8)	18(21.2)	78(19.0)	24(1.2)	544(23.6)	1577(25.4)	
* BRCA1*+2	1 (0.8)	11(0.6)	0	0	0	0	0	0	0	11(0.7)	23(0.4)	
* BRCA1*or2	0	22(1.2)	0	0	12(6.3)	0	0	0	1(0.0)	20(1.4)	55(0.9)	
Mean year of birth	1961.6	1959.3	1965.2	1951.3	1953.6	1957.6	1958.2	1960.6	1963.3	1959.4	1960.5	<10^−4^
Range	1931–82	1917–89	1942–82	1935–68	1920–74	1933–74	1933–76	1921–83	1923–88	1916–87	1916–89	
Mean age at baseline interview	42.3	46.0	45.2	47.2	46.4	43.0	42.8	42.1	43.6	45.3	44.7	<10^−4^
Range	25–68	25–79	27–67	30–62	25–79	25–66	25–68	25–78	25–79	25–78	25–79	
Subjects with breast cancer number (%)	56(44.1)	774(43.5)	22(73.3)	26(86.7)	86(44.6)	22(52.4)	30(35.3)	53(13.0)	872(42.5)	692(47.0)	2633(42.3)	<10^−4^
Mean age at diagnosis	39.9	43.1	38.7	42.4	42.8	40.0	40.5	44.5	44.1	41.8	42.9	
Range	27–63	19–75	26–60	25–60	20–78	29–58	30–53	31–68	24–77	21–74	19–78	<10^−4^
Mean year at baseline interview	2003.9	2005.2	2010.4	1998.6	2000.1	2000.6	2000.8	2002.7	2006.8	2004.7	2005.2	<10^−4^
Range	1999–08	1994–15	2009–11	1997–01	1996–02	1997–05	2000–02	1991–10	1999–15	1994–15	1991–15	
Mean year at follow	2011.0	2013.8	2014.5	2009.2	2005.4	2006.4	2005.2	2010.5	2013.6	2012.1	2012.7	<10^−4^
Range	2004–13	1999–13	2012–17	2004–12	2001–10	2004–17	2004–06	2002–13	2002–17	1998–17	1998–17	
Mean age at follow-up	49.4	54.6	49.3	57.9	51.7	48.8	47.1	49.9	50.4	52.8	52.1	<10^−4^
	29–81	27–96	29–71	37–74	28–83	30–84	28–71	28–87	27–86	27–88	27–96	
Mean years of follow-up	7.1	8.6	4.1	10.6	5.3	5.8	4.3	7.8	6.7	7.4	7.5	<10^−4^
Range	1.6–13.3	15–21.5	2.0–7.6	3.1–14.6	1.7–11.1	2.0–17.7	2.0–5.8	1.8–18.1	1.5–17.0	1.5–22.2	1.5–22.2	

^a^ANOVA for differences in mean values between the ten countries; chi-square test for the differences in frequency distributions of the nine countries

### Bilateral prophylactic mastectomy

**Fig. 1:**
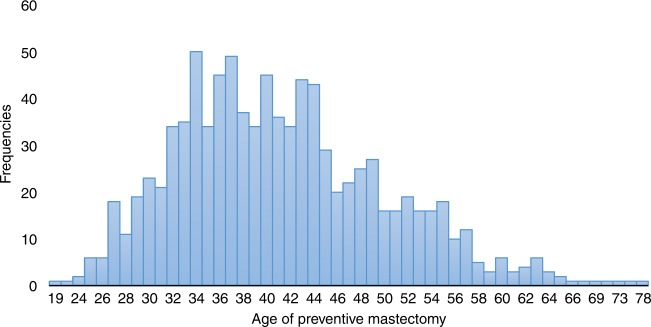
The distribution of age of preventive mastectomy among subjects without breast cancer

Three-thousand four-hundred and thirteen women had no history of breast cancer at any time and had provided data on bilateral prophylactic mastectomy. Of these women, 950 (27.8%) had a prophylactic bilateral mastectomy (Table 2). The mean age at prophylactic mastectomy was 41.8 years (range 19–78 years). The distribution in ages of prophylactic mastectomy are presented in Fig. 1. The mean age at mastectomy was 40.7 years for *BRCA1* carriers and was 42.4 years for *BRCA2* carriers, and 3.4% of the mastectomies were done at age 60 and above. The mastectomy rate was highest in the United States (49.9%) and lowest in Poland (4.5%). Women who received genetic testing in 2009 or later were more likely to elect for prophylactic mastectomy compared to women who received testing prior to 2009 (30.3% versus 26.9%) (*P* = 0.04) (Table 3). The increase was restricted to the United States (56.8% versus 46.4%); among Canadian women there was a slight decline (35.9% versus 39.1%) and the rates in Poland were uniformly low across all decades.

**Table 2:** Uptake of options by country

Variables	Austria	Canada	China	France	Israel	Italy	Holland	Norway	Poland	USA	All
	N^1^ = 127	N^1^ = 1780	N^1^ = 30	N^1^ = 30	N^1^ = 193	N^1^ = 42	N^1^ = 85	N^1^ = 408	N^1^ = 2054	N^1^ = 1474	N^1^ = 6223
	N^2^ = 71	N^2^ = 1005	N^2^ = 8	N^2^ = 4	N^2^ = 107	N^2^ = 20	N^2^ = 55	N^2^ = 187	N^2^ = 1182	N^2^ = 774	N^2^ = 3413
	N^3^ = 51	N^3^ = 623	N^3^ = 5	N^3^ = 3	N^3^ = 102	N^3^ = 18	N^3^ = 37	N^3^ = 107	N^3^ = 1129	N^3^ = 388	N^3^ = 2463
Oophorectomy^1^											
All	77(60.6)	1278(71.8)	11(36.7)	25(83.3)	130(67.4)	22(52.4)	55(64.7)	260(63.7)	1041(50.7)	1124 (76.3)	4023(64.7)
<2009	77(60.6)	914(73.5)	NA	25(83.3)	130(67.4)	22(52.4)	55(64.7)	252(65.5)	785(52.4)	814(77.5)	3074(66.1)
>=2009	0	364(67.9)	11(36.7)	NA	NA	NA	NA	8(34.8)	256(46.0)	310(73.1)	949(60.5)
PM^2^											
All	20(28.2)	382(38.0)	3(37.5)	1(25.0)	5(4.7)	2(10.0)	18(32.7)	80(42.8)	53(4.5)	386(49.9)	950(27.8)
<2009	20(28.2)	259(39.1)	NA	1(25.0)	5(4.7)	2(10.0)	18(32.7)	76(41.5)	33(4.0)	244(46.4)	658(26.9)
>=2009	0	123(35.9)	3(37.5)	NA	NA	NA	NA	4(100)	20(5.6)	142(56.8)	292(30.3)
Mammography^3^											
All	51(100)	599(96.5)	5(100)	3(100)	98(96.1)	18(100)	37(100)	103(98.1)	731(64.8)	374(96.5)	2019(82.2)
<2009	51(100)	392(97.8)	NA	3(100)	98(96.1)	18(100)	37(100)	103(98.1)	564(71.4)	269(96.1)	1535(85.9)
>=2009	0	207(94.1)	5(100)	NA	NA	NA	NA	NA	167(49.4)	105(97.2)	484(72.1)
MRI^3^											
All	45(88.2)	465(76.7)	5(100)	3(100)	3(3.0)	14(77.8)	35(94.6)	93(93.0)	835(74.0)	252(71.2)	1750(72.8)
<2009	45(88.2)	276(71.5)	NA	3(100)	3(3.0)	14(77.8)	35(94.6)	93(93.0)	574(72.7)	161(65.5)	1204(69.5)
>=2009	0	189(85.9)	5(100)	NA	NA	NA	NA	NA	261(77.0)	91(84.3)	546(81.3)
Tamoxifen/Raloxifene^3^											
All	0	64(10.3)	0	0	10(9.8)	0	0	0	24(2.1)	57(14.7)	155(6.3)
<2009	0	45(11.2)	NA	0	10(9.8)	0	0	0	22(2.8)	44(15.7)	121(6.8)
>=2009	0	19(8.6)	0	NA	NA	NA	NA	NA	2(0.6)	13(12.0)	34(5.1)

^1^All subjects

^2^Subjects without breast cancer; 177 subject with missing data on mastectomy excluded;

^3^Subjects without breast cancer and without prophylactic mastectomy; 5 subject with missing data on mammography; 59 missing MRI.

**Table 3:** Prevention options by year of enrolled to the study (baseline questionnaire filled), all countries included

Measures	Before 2009	After/at 2009	
	N = 4653	N = 1570	P-value^4^
Oophorectomy^1^			
No	1579(33.9)	621(39.6)	
Yes	3074(66.1)	949(60.5)	<0.0001
Oophorectomy (over age 35 at FU)^1^	(N = 4446)	(N = 1319)	
No	1380(31.0)	377(28.6)	
Yes	3066(69.0)	942(71.4)	0.09
PM^2^			
No	1791(73.1)	672(69.7)	
Yes	658(26.9)	292(30.3)	0.04
Tamoxifen/Raloxifene^3^			
No	1670(93.2)	638(94.9)	
Yes	121(6.8)	34(5.1)	0.12
MRI^3^			
No	528(30.5)	126(18.7)	
Yes	1204(69.5)	546(81.3)	<0.0001
Mammography^3^			
No	252(14.1)	187(27.9)	
Yes	1535(85.9)	485(72.2)	<0.0001

^1^All subjects

^2^Subjects without breast cancer;

^3^Subjects without breast cancer and without prophylactic mastectomy;

^4^Chi-square test

### Prophylactic bilateral salpingo-oophorectomy (BSO)

**Fig. 2:**
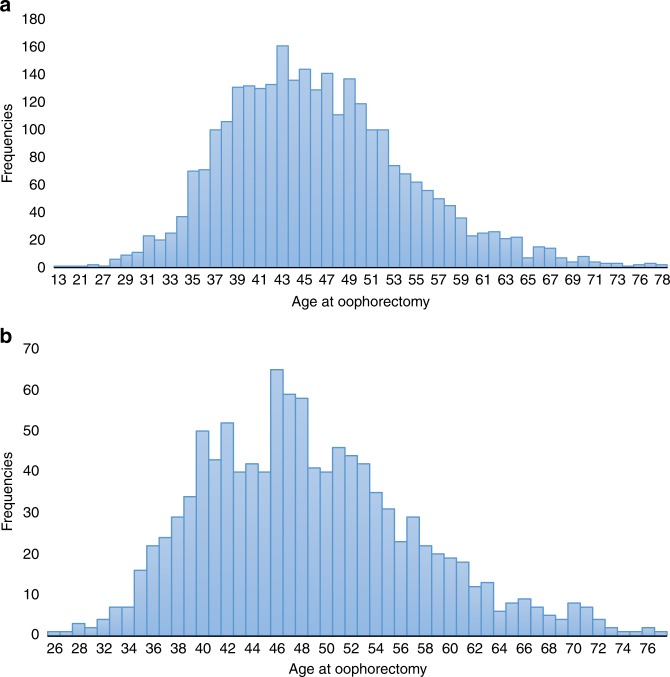
The distribution of age of preventive oophorectomy, **a** BRCA1 subjects, **b** BRCA2 subjects

Four-thousand twenty-three (64.7%) of the *BRCA* mutation carriers had a BSO, including 62.8% of the *BRCA1* carriers and 69.7% of the *BRCA2* carriers. Of the 2634 women with a previous diagnosis of breast cancer, 1862 women (70.7%) had a BSO after breast cancer diagnosis. For women without breast cancer the mean age at BSO was 45.6 years (range 13–78 years), 44.7 years for *BRCA1* carriers and 47.7 years for *BRCA2* carriers. The distribution of ages of preventive oophorectomy by mutation is in Fig. 2. Among *BRCA1* carriers, 7.2% of the oophorectomies were done at age 35 or before. Among *BRCA2* carriers, 37.8% of the oophorectomies were done at age 45 or before. In women over the age of 35 years at last follow-up, uptake was 69.5% and there were no significant differences in uptake between women who received genetic testing before 2009 (69.0%) and those who were tested in 2009 or later (71.4%; *P* = 0.09) (Table 3). Uptake was highest in France (83.3%) and the lowest in China (36.7%).

### Chemoprevention

Of the 2463 women without a history of breast cancer or prophylactic mastectomy, 155 (6.3%) took tamoxifen or raloxifene for prevention. The rate ranged from 2% in Poland to 15% in the USA. In the USA, 12.7% of BRCA1 carriers and 17.4% of BRCA2 carriers elected for chemoprevention (*P* = 0.20). Overall, there was no temporal change in the use of chemoprevention (6.8% of women with a baseline before 2009 and 5.1% in women with a baseline of 2009 or later).

### Breast screening

Overall, the uptake of mammography was 82.1%, but this decreased with time; women receiving genetic testing in 2009 or later were less likely to have a mammogram than women tested prior to 2009 (72.1% versus 85.9%) (*p* < 0.0001). To a large extent this was due to the increasing trend in MRI in Poland (72.7% versus 77.0%) coupled with a decline in mammography in the same country (71.4% versus 49.4%). Screening with breast MRI increased over time in Canada, Poland and the United States, overall women receiving testing in 2009 or later were significantly more likely to have a breast MRI compared to women tested prior to 2009 (81.3% versus 69.5%; *p* < 0.0001). Poland is the only country where the use of MRI now exceeds the use of mammography.

## Discussion

Genetic testing for *BRCA1* and *BRCA2* was initiated in 1995 and has continued to expand throughout the past two decades. Reasons for expansion include an increase in the number of laboratories offering testing combined with a decrease in cost, celebrity endorsement and increasing evidence for the clinical benefit of knowing one’s mutation status. In 1995, we initiated a long-term follow-up study to investigate, among other topics, patient decisions about preventive options. In 2008, we reported on the uptake rates of various cancer risk-reducing options in 2677 women from nine countries.^20^ In the past 10 years, evidence has accumulated regarding the effectiveness of MRI-based screening, preventive mastectomy and preventive salpingo-oophorectomy in women with a *BRCA* mutation.^3,4^^5–11^ In this updated analysis of 6223 female *BRCA* carriers from ten countries, we report that there has been little increase in the rates of preventive oophorectomy among those with a positive result, but there has been a significant increase in the uptake of bilateral prophylactic mastectomy in women who received genetic testing after 2008. There are persistent differences in uptake of cancer risk-reduction options by country.

The National Comprehensive Cancer Network (NCCN) provides guidelines for the management of women with a *BRCA1* or *BRCA2* mutation.^2^ Prophylactic mastectomy and chemoprevention are both options for cancer risk reduction and should be discussed. However, it is recommended that women have a BSO between the ages of 35 and 40 years, when childbearing is complete. Previous research has shown that BSO reduces the risk of ovarian cancer,^3,4,21^ and decreases all-cause mortality in women with a *BRCA* mutation by 77%.^4^ In the current study, 69.5% of women over the age of 35 years had a BSO. When we only included women over the age of 40 years, 74.6% had BSO. Very few women with a *BRCA1* mutation had an oophorectomy prior to age 35. The mean age of BSO was 45 years for *BRCA1* carriers, and 48 years for *BRCA2* carriers. Ideally, women would have BSO at a younger age, prior to the age in which the incidence of ovarian cancer starts to increase. This late age of uptake may not reflect women’s decisions, buy may reflect the age in which genetic testing is performed.

Bilateral prophylactic mastectomy is an option for unaffected *BRCA* mutation carriers and has been shown to reduce breast cancer incidence.^15–19^ In our international cohort, 27.9% of the unaffected *BRCA* mutation carriers had bilateral prophylactic mastectomy, however, uptake varied greatly between countries with the highest uptake in the United States (49.9%) and the lowest uptake in Poland (4.5%). Previous research in single countries has reported uptake rates as high as 51% in the Netherlands^22^ to a low of 5% in France,^23^ with uptakes of 40% in the United Kingdom^24^ and 21% in Australia.^25^ These discrepancies in uptake could be due to differences in physician’s attitudes by country, which has been shown to exist. In recent research, both general physicians and surgeons from France and Germany reported significantly less-positive attitudes towards prophylactic mastectomy compared to those in the Netherlands and the United Kingdom.^26^ Furthermore, uptake of prophylactic mastectomy could change in the future as more evidence becomes available on the effectiveness of breast MRI screening in BRCA carriers.

In addition to differences in uptake of prophylactic mastectomy by country, there were also differences in uptake according to when a woman received genetic testing. Women who received genetic testing more recently (in 2009 or later) were significantly more likely to have a bilateral prophylactic mastectomy (30.3%) compared to women who received genetic testing prior to 2009 (26.9%) (*P* = 0.04). Since 2013, when Angelina Jolie disclosed her *BRCA* status and her choice to undergo preventive surgeries, referrals for genetic testing and preventive surgeries have increased worldwide.^27–29^ In the United Kingdom, there was a 2.5-fold increase in uptake of bilateral prophylactic mastectomy in the 6–24 months following Jolie’s disclosure.^27^ In addition, in recent years, alternative surgical options have been available for *BRCA* mutation carriers, including nipple-sparing mastectomy in which the nipple-areolar complex is preserved. The use of this surgery in *BRCA* mutation carriers was controversial, however, there is growing evidence that this surgery is oncologically safe. In a recent multi-institutional study of 202 unaffected *BRCA* mutation carriers who underwent a bilateral nipple-sparing prophylactic mastectomy, no breast cancer events occurred at any site in the 62 months of follow-up.^30^ In addition to the demonstrated oncologic safety of the surgery, it has also been shown to optimise cosmesis, and patients report higher levels of psychosocial and sexual well-being.^31^

Previous research has demonstrated that annual magnetic resonance imaging (MRI) of the breasts is significantly more sensitive compared to annual mammography.^5–11^ International guidelines, including the NCCN and NICE (National Institute for Health and Care Excellence) guidelines recommend annual breast MRI starting at age 30. In an international survey of 22 high-risk clinics from 16 countries, all clinics reported that their breast screening recommendations included at least annual MRI, although ages at initiation varied.^32^ In the current study, 72.8% of women reported having received a breast MRI within the previous year. However, those who received genetic testing in 2009 or later, were significantly more likely to have a breast MRI (81.3%) compared to women who received testing earlier than 2009 (69.5%) (*p* < 0.0001). We saw a decline in mammogram use among along with an increase in MRI screening. The global decline was entirely due to women in Poland foregoing mammography since 2009, in Canada and the USA the great majority of carriers continued with regular mammography screening. Among women who undergo regular MRI screening there does not appear to be an incremental advantage to doing mammography as well.^33^

Overall, uptake of both breast MRI screening and BSO is not optimal in this cohort of *BRCA* carriers from around the world. In order to minimise the risk of cancer incidence and mortality in women with a *BRCA1* or *BRCA2* mutation, uptake of both breast MRI and BSO should approach 100%. For many women with a *BRCA* mutation, after receiving genetic test results, follow-up care is coordinated through non-specialised primary-care providers. Alternative models of care for long-term follow-up of *BRCA* mutation carriers need to be considered. In Israel, a dedicated follow-up clinic for *BRCA* carriers has been established and provides multidisciplinary care to support the medical and emotional needs of this high-risk population. Within a median follow-up of 46 months, 99.4% of patients over the age of 40 years had a BSO, and 17 patients were diagnosed with invasive breast cancer (16 of which were Stage I).^33^ This suggests that more specialised follow-up care for *BRCA* mutations may result in superior outcomes and should be considered moving forward.

There are several limitations to our study. We have included patients from clinical centres in which we have collaborations, and they may not represent uptake across each individual country. However, many of these collaborating sites are academic clinical sites, and we may expect that follow-up care could be more specialised than what may exist for patients who receive direct to consumer genetic testing, or genetic testing in a non-specialised environment. In addition, for some of the countries included in this study, the number of patients was small, and may not represent the population of *BRCA* carriers in the respective countries. For five of the ten countries information was available for only the first period (1995 to 2008) and thus the trend estimates for the overall cohort are not reliable. Also, the size of the country cohorts varied widely, and we present the crude data on 6226 women, not weighted according to underlying population size. For this reason, the most reliable information on trends comes from the individual countries and not from the aggregate data (Table 2).

Overall, many women with a *BRCA* mutation are electing for cancer surveillance or prevention. For women who received genetic testing more recently, uptake of both bilateral prophylactic mastectomy and breast MRI is significantly higher than those who received genetic testing more than 10 years ago. However, uptake of both BSO and breast MRI is not optimal, and interventions to increase uptake are needed. By increasing uptake of BSO and breast MRI, cancer incidence and mortality in women with a *BRCA1* or *BRCA2* mutation could be reduced.

## Data Availability

Data supporting the results reported in this article may be requested from the corresponding author.
